# Spatial Distribution of Tsetse Flies and Trypanosome Infection Status in a Vector Genetic Transition Zone in Northern Uganda

**DOI:** 10.1155/2022/9142551

**Published:** 2022-06-01

**Authors:** Robert Opiro, Okello Allele Moses, Robert Opoke, Francis A. Oloya, Esther Nakafu, Teresa Iwiru, Richard Echodu, Geoffrey M. Malinga, Joel L. Bargul, Elizabeth A. Opiyo

**Affiliations:** ^1^Department of Biology, Faculty of Science, Gulu University, P.O. Box 166, Gulu, Uganda; ^2^Gulu University Multifunctional Research Laboratories, P.O. Box 166, Gulu, Uganda; ^3^Department of Biology, Faculty of Science, Muni University, P.O. Box 725, Arua, Uganda; ^4^Department of Molecular Biology, College of Veterinary Medicine, Animal Resources and Biosecurity, Makerere University, P.O. Box 7062, Kampala, Uganda; ^5^International Centre for Insect Physiology and Ecology (ICIPE), Nairobi, Kenya

## Abstract

**Background:**

Tsetse flies are vectors of the genus Trypanosoma that cause African trypanosomiasis, a serious parasitic disease of people and animals. Reliable data on the vector distribution and the trypanosome species they carry is pertinent for planning sustainable control strategies. This study was carried out to estimate the spatial distribution, apparent density, and trypanosome infection rates of tsetse flies in two districts that fall within a vector genetic transition zone in northern Uganda.

**Materials and Methods:**

Capturing of tsetse flies was done using biconical traps deployed in eight villages in Oyam and Otuke, two districts that fall within the vector genetic transition zone in northern Uganda. Trapped tsetse flies were sexed and morphologically identified to species level and subsequently analyzed for detection of trypanosome DNA. Trypanosome DNA was detected using a nested PCR protocol based on primers amplifying the internal transcribed spacer (ITS) region of ribosomal DNA.

**Results:**

A total of 717 flies (406 females; 311 males) were caught, all belonging to the *Glossina fuscipes fuscipes* species. The overall average flies/trap/day (FTD) was 2.20 ± 0.3527 (mean ± SE). Out of the 477 (201 male; 276 females) flies analyzed, 7.13% (34/477) were positive for one or more trypanosome species. Three species of bovine trypanosomes were detected, namely, *Trypanosoma vivax*, 61.76% (21/34), *T. congolense*, 26.47% (9/34), and *T. brucei brucei*, 5.88% (2/34), and two cases of mixed infection of *T. congolense* and *T. brucei brucei*, 5.88% (2/34). The infection rate was not significantly associated with the sex of the fly (generalized linear model (GLM), *χ*^2^ = 0.051, *p* = 0.821, df = 1, n = 477) and district of origin (*χ*^2^ = 0.611, *p* = 0.434, df = 1, *n* = 477). However, trypanosome infection was highly significantly associated with the fly's age based on wing fray category (*χ*^2^ = 7.56, *p* = 0.006, df = 1, *n* = 477), being higher among the very old than the young.

**Conclusion:**

The relatively high tsetse density and trypanosome infection rate indicate that the transition zone is a high-risk area for perpetuating animal trypanosomiasis. Therefore, appropriate mitigation measures should be instituted targeting tsetse and other biting flies that may play a role as disease vectors, given the predominance of *T. vivax* in the tsetse samples.

## 1. Introduction

Tsetse flies (Diptera: Glossinidae), vector trypanosome parasites that cause both human African trypanosomiasis (HAT) and animal African trypanosomiasis (AAT). The diseases impose a severe burden on human and livestock health in sub-Saharan Africa [1]. Thirty-seven countries are affected, with more than 50 million cattle, 70 million small ruminants [2], and 55 million people at risk of contracting the diseases [3]. The Food and Agricultural Organization (FAO) of the United Nations estimates that Africa loses up to US$1.5 billion annually as a result of the disease [4]. Thus, these diseases, together, have both health and social impacts.

Over 70% (140,000 km^2^) of Uganda's land area is estimated to be infested by tsetse flies, but with varying levels of prevalence [5], with *Glossina fuscipes fuscipes* being the most predominant [6]. Recent population genetic studies on *G. f. fuscipes* in northern Uganda identified a vector “genetic transition zone” where different lineages freely mix and interbreed [7]. This zone also coincides with a previously known HAT disease-free belt in northern Uganda, where the two forms of HAT are likely to merge [7, 8]. The zone, therefore, presents an epidemiologically significant area due to the existence of the two forms of human-infective trypanosomes (*Trypanosoma brucei rhodesiense* and *Trypanosoma brucei gambiense*), parasite reservoirs, vectors, and susceptible hosts. Yet there is a paucity of data on the vector distribution and parasite infection status to guide control interventions. We assessed the apparent density and tsetse trypanosome infection status in Oyam and Otuke districts that fall within the genetic transition zone.

## 2. Materials and Methods

### 2.1. Study Area

**Figure 1:**
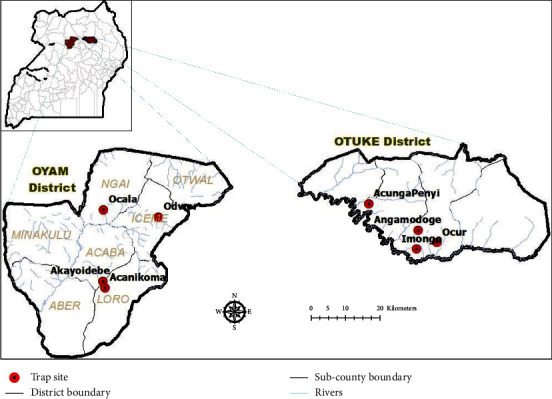
Map of study area showing trapping sites. In the upper left hand corner is the inset map of Uganda showing the location of the two surveyed districts, while below it are the maps of the two districts showing the sampling sites.

The study was conducted in Oyam (2.2776°N, 32.4467°E) and Otuke (2.4444°N, 33.5053°E) districts, located within the vector genetic transition zone in northern Uganda [7] (Figure 1). Flat grasslands and seasonally flooded swamps characterize both districts. Average annual rainfall varies between 1200 and 1600 mm, peaking in April-May and in August-October. The average minimum and maximum temperatures are 22.5°C and 25.5°C, respectively. Subsistence agriculture is the main economic activity. Livestock reared comprise goats, sheep, cattle, chickens, and turkeys.

### 2.2. Entomological Survey

Ninety-eight biconical traps [9] were deployed along suitable habitats, targeting areas of human and animal activities in eight villages within the two districts in the period between April and May 2019. After recording the coordinates, traps were left in the field for at least 72 hours. Captured flies were identified morphologically, counted, and sorted into males and females and into teneral and nonteneral status [10]. Approximate ages of the flies were estimated using wing fray analyses [11]. For statistical analyses, the flies were categorized based on wing fray as “young tsetse” (WF1-2), “old tsetse” (WF 3-4), and “very old tsetse” (WF 5-6). Nonteneral flies were preserved in 70% ethanol in sealed Eppendorf tubes for subsequent DNA extraction and PCR assays.

### 2.3. Infection Rates and Trypanosome Species Determination

#### 2.3.1. DNA Extraction

DNA was extracted from the whole tsetse body using the PureLink™ extraction kit from Invitrogen, following the manufacturer's instructions (https://www.thermofisher.com). The supernatant was used either directly for PCR or stored at -20°C until use.

#### 2.3.2. PCR Amplification of DNA

PCR examinations were done on 66.5% (477/717) of the captured flies. Extracted DNA was amplified following the nested PCR protocol of Cox et al. [12], using the same primer sequences but with slight modifications in amplification conditions. The outer primer sequences were ITS1 (5-GAT TAC GTC CCT GCCATT TG-3) and ITS2 (5-TTG TTC GCT ATC GGTCTT CC-3), and the inner primer sequences were ITS3 (5-GGA AGC AAA AGT CGT AACAAG G-3) and ITS4 (5-TGT TTT CTT TTC CTCCGC TG-3) [13]. The PCR amplifications were performed in a total reaction volume of 20 *μ*l containing 10 pmol of each primer and 2 *μ*l of each DNA template. The reaction conditions followed the protocol outlined in [12] including 1 cycle of initial denaturation at 95°C for five minutes, followed by 40 amplification cycles each consisting of one minute denaturation at 94°C, one minute primer annealing at 55°C, and two minutes polymerization at 72°C, with a final extension at 72°C for five minutes. For the second round of reaction, 2 *μ*l of the PCR product from the first round was placed in a fresh tube and 20 *μ*l of the reaction mixture was added as detailed for the outer primers, with the exception of the substitution of the outer primers (ITS1 and ITS2) with the inner primers (ITS3 and ITS4) and with the same conditions as in round one. Negative controls in which DNA templates were replaced with sterile water were used to minimise bias due to false positives during repeated PCRs. Positive control DNAs (of trypanosome species) were included in all the PCR reactions. The reactions were carried out in a highly sterilized and dedicated PCR diagnostics lab environment at the Gulu University Multifunctional Research Laboratories (GUMRL). The PCR products (5 *μ*l) were then loaded into a casted 1.8% agarose gel stained with gel red with a 100 bp gene marker. These were run in an electrophoresis gel tank for 45 minutes at 100 V in 0.5X TBE buffer. The amplified products were visualized on a gel to identify the trypanosome species/subspecies by referencing previously documented sizes [12]. Where the band sizes corresponded to *Trypanosoma brucei*, confirmation of subspecies was done by running a second PCR for diagnosis of *T. b. gambiense* employing a nested PCR with a first reaction using TgsGP1/2 primers [14] and a second one with TgsGP sense2/antisense2 primers described by Morrison et al. [15].

### 2.4. Data Analyses

Apparent density was expressed as the number of flies per trap per day (flies/trap/day (FTD)) using the formula FTD = *ΣF*/*T* × *D*, where *ΣF* is the total number of tsetse flies caught, *T* is the number of traps deployed, and *D* is the number of days of trapping in the field [16]. Tsetse infection rates were calculated by dividing the number of flies infected with trypanosomes by the total number of flies analyzed using PCR and expressed as percentages. To identify predictors of tsetse infection, a generalized linear model (GLM) was fitted with a negative binomial error distribution and a log link function (tsetse infection as the response variable and the fly's sex, district, and age as covariates).

## 3. Results

### 3.1. Entomological Data

A total of 717 tsetse flies were caught, all belonging to the *Glossina fuscipes fuscipes* species. More females (56.62%; 406/717) than males (43.38%; 311/717) were caught in the traps (Table 1). The average FTD for all sites combined was 2.20 ± 0.35 (mean ± SE), with the highest being in Akayoidebe in Oyam and the least in Imongo in Otuke (Table 1). However, the average FTD between the two districts did not differ significantly (Mann–Whitney *U* test, *U* = 4.0, *p* = 0.248). The average mean wing fray value (MWFV) for all flies in both districts was 2.90 ± 0.08, corresponding to an average estimated age of 22 days, with the ages for populations in the different sites varying from 19 to 25 days. The village with the oldest flies (25 days; MWFV 3.3) was Imongo and the one with the youngest flies (19 days; MWFV 2.6) was Ocur in Otuke district (Table 1).

### 3.2. Trypanosome Infection Rates and Species

The overall infection rate was 7.13% (34/477). The prevalence of trypanosome infection was not significantly associated with the fly's sex (GLM, *χ*^2^ = 0.051, *p* = 0.821, df = 1, *n* = 477) and district of origin (*χ*^2^ = 0.611, *p* = 0.434, df = 1, *n* = 477). However, the trypanosome infection rate was significantly associated with the fly's age based on the wing fray category (*χ*^2^ = 7.56, *p* = 0.006, df = 1, *n* = 477), and the prevalence of infection was higher among the very old than the young tsetse. Akayoidebe village in Oyam district had the highest overall prevalence of infected flies (10/34), while there was no infected fly in Angamodoge in Otuke district (Table 2). The village with the highest infection rate in Otuke was Ocur (4/10).

PCR examination detected three species of trypanosomes; *T. vivax* (21/34), *T. congolense* (9/34), and *T. brucei* (2/34). Species-specific PCR later confirmed the presence of *T. brucei* as *T. brucei brucei* in all cases. There were two cases of mixed infections of *T. vivax*/*T. brucei* detected in Acungapenyi and Akayoidebe villages in Otuke district (Table 2).

## 4. Discussion

Our findings showed *G. f. fuscipes* as the only tsetse species in the study area, which agrees with the habitat suitability prediction of tsetse species in Uganda, showing this area as a predominantly *G. f. fuscipes* belt [17, 18]. The vast majority of traps in our sampling scheme were placed along river systems and wetlands that are the expected habitats of *G. f. fuscipes* [19–21]. Nevertheless, other traps set in peridomestic environments and anthropised landscapes disrupted through cultivation and clearance of bushes also caught *G. f. fuscipes*, confirming its ability to adapt and breed in peridomestic habitats [22].

Apparent density (FTD) in the different sites sampled varied from 0.78 to 4.29, which is comparable to those found in a previous study in midnorthern Uganda [23] and in northwestern Uganda [24, 25]. Compared to those reported in southeastern Uganda [26], the low apparent density in the present study could be explained by differences in some bioclimatic conditions between the north and south of Uganda [27]. However, the lack of variation in apparent density per trap between sites is probably linked to the similarity in the tsetse biotopes between the different areas sampled. The study area is uniformly characterized by uninterrupted suitable habitats along major rivers, streams, and wetlands comprised of bushy shrubs and thickets of wetland plants.

The results of molecular analysis using PCR revealed a prevalence rate of 7.13% of *Trypanosoma* infection, higher than those reported in earlier studies by Azabo et al. [23] in midnorthern Uganda, Waiswa et al. [26] in southeastern Uganda (5.6%), and Adungo et al. [28] in Busia, Kenya (4.17%). The relatively high infection rates reported in tsetse flies indicate ongoing transmission of African trypanosomiasis in animals in the study area. Only three species of trypanosomes, namely, *Trypanosoma. vivax*, *T. congolense*, and *T. brucei brucei*, were detected in the present study, despite the capability of ITS PCR to detect a wide range of other trypanosomes like *T. theileri* and *T. simiae* [12]. This indicates the important role these detected species currently play in the epidemiology of AAT in the study area. However, the study area was small compared to the size of the transition zone (>200 km); thus, some infections could have been missed.

The most prevalent trypanosome species was *T. vivax*, followed by *T. congolense* and *T. brucei brucei.* The predominance of *T. vivax* corroborates other findings in the northern and Karamoja regions of Uganda [29, 30]. Moreover, this suggests that other biting flies other than tsetse could also be playing a role in mechanical transmission. The role of mechanical vectors in the transmission of African livestock trypanosomes has always been controversial relative to tsetse flies, their cyclical vectors. However, experimental works successfully demonstrated that African tabanids mechanically transmit *T. vivax* to cattle [29].

Though no human infective trypanosome species were detected in the study, the result should be interpreted with caution because sampling was done in only two of the over 10 districts in the transition zone. To better understand the epidemiology of HAT in the area, we recommend a more extensive screening of the tsetse vectors and parasite reservoir animals covering the entire transition zone.

Results showed that tsetse infection rate was not associated with sex, unlike in other previous studies where fly sex influenced susceptibility to trypanosome infection, with females being more susceptible to parasite infection than males [31]. However, other studies have shown the contrary [32]. Thus, the precise associations of trypanosome infection with sex remain unresolved and require further investigations. Infection rates, however, were strongly associated with age, with older flies having a higher level of infection than their younger counterparts, agreeing with the observation that some trypanosomes require a longer time for maturation, e.g., *Trypanosoma. congolense* takes up to 15–20 days at 24°C, while *T. brucei* takes even longer [19]. Thus, older flies are expected to have more infections than young flies.

In terms of implications for control, the study generally shows that trypanosomiasis is a significant threat in the study area and interventions should be initiated or strengthened. Continental initiatives such as the Pan African Tsetse and Trypanosomiasis Eradication Campaign (PATTEC) should be embraced and rolled out to all areas in the Country. In Uganda, phase I of the PATTEC initiative was restricted only to the Lake Victoria basin, infested with *Glossina fuscipes fuscipes* (*Gff*). This has left areas such as northern Uganda devoid of extensive control measures. A prolonged period of civil unrest in the region led to a disruption of control efforts and a breakdown in social infrastructure, and later control activities were decentralized and are currently managed by underfunded district health and veterinary authorities [33].

## 5. Conclusions

The relatively high tsetse density and trypanosome infection rates imply that the transition zone is a bovine trypanosomiasis high-risk area that necessitates the institution of appropriate risk mitigation measures. The high prevalence of *Trypanosoma vivax* suggests that the mechanical transmission of trypanosomiasis could occur in the area. Therefore, control interventions should not only concentrate on reducing tsetse density but target other biting insects like stomoxys and tabanids that possibly play a role in transmission. Finally, although human infective trypanosome infections were not detected, future studies should be aimed at extensively sampling the entire transition zone.

## 6. Limitations of the Study

1. The study did not assess the trypanosome infection status in animal reservoirs, which could have given a complete picture of the disease burden in the study area
2. The study was cross-sectional in design and this did not allow for evaluation of the effect of seasonality on the distribution of both the vectors and the parasites. Future studies should be aimed at assessing the effect of seasonality since seasons influence the climatic drivers that create fluctuations in tsetse fly populations

## Figures and Tables

**Table 1:** Entomological data of tsetse captured in Oyam and Otuke districts.

District	Village	Sampling period	*X*	*Y*	No traps	Male	Female	FTD	MWFV	Estimated age (days)
Otuke	Ocur	April 2019	2.37131	33.346985	12	32	65	2.69	2.6	19
	Imongo	April 2019	2.344123	33.28905	9	14	7	0.78	3.3	25
	Angamodoge	April 2019	2.387388	33.312853	9	11	23	1.26	2.7	20
	Acungapenyi	April 2019	2.468952	33.190915	11	34	37	2.15	2.9	22
Oyam	Akayoidebe	May 2019	2.26922	32.52208	15	83	110	4.29	3.0	23
	Ocala		2.42749	32.528071	10	29	36	2.17	3.1	24
	Acanikoma	May 2019	2.24883	32.528071	20	87	89	2.60	2.9	22
	Odwor	May 2019	2.44776	32.66049	12	21	39	1.67	2.7	20
Total					**98**	**311**	**406**			

FTD refers to the number of flies caught per trap per day for the different sites; MWFV is the mean wing fray value while *X* and *Y* refers to the *X* and *Y* coordinates (latitude and longitude) of the villages.

**Table 2:** Trypanosome infection rate and species in Oyam and Otuke districts.

District	Village	Examined	Total infected	Infected male	Infected female	*T. vivax*	*T. congolense*	*T. brucei*	Mixed
Otuke	Ocur	60	4	1	3	3	1	0	0
	Imongo	14	1	0	1	1	0	0	0
	Angamodoge	21	0	0	0	0	0	0	0
	Acungapenyi	48	7	1	6	4	2	0	1
Oyam	Akayoidebe	130	10	7	3	5	2	2	1
	Ocala	43	3	1	2	2	1	0	0
	Acanikoma	116	6	4	2	5	1	0	0
	Odworo	45	3	1	2	1	2	0	0
Total		**477**	**34**	**15**	**19**	**21**	**9**	**2**	**2**

## Data Availability

The datasets analyzed during the current study are available from the corresponding author upon reasonable requests.

## Abbreviations

AAT: African animal trypanosomiasis
HAT: Human African trypanosomiasis
FTD: Flies per trap per day
GLM: Generalized linear model
ITS: Internal transcribed spacer.
